# Supplementary material for: Data-optimal scaling of paired antibody language models
Source: bioRxiv. 2025 Nov 4:2025.09.02.673765. Originally published 2025 Sep 6. Preprint. [Version 2] doi: 10.1101/2025.09.02.673765 (PMC12424686; doi:10.1101/2025.09.02.673765)
Supplement: Supplement 1 [file NIHPP2025.09.02.673765v2-supplement-1.pdf]

bioRxiv preprint doi: https://doi.org/10.1101/2025.09.02.673765; this version posted November 5, 2025. The copyright holder for this preprint (which was not certified by peer review) is the author/funder, who has granted bioRxiv a license to display the preprint in perpetuity. It is made available under aCC-BY 4.0 International license.

SUPPLEMENTARY INFORMATION

Model size (M)	Dataset	Checkpoint step	Transformer layers	Attention heads	Hidden size	Intermediate size
8	F	500,000	6	20	320	1280
	H	435,000				
	Q	425,000				
35	F	500,000	12	20	480	1920
	H	430,000				
	Q	240,000				
150	F	500,000	30	20	640	2560
	H	330,000				
	Q	165,000				
350	F	500,000	32	20	960	3840
	H	300,000				
	Q	155,000				
650	F	395,000	33	20	1280	5120
	H	330,000				
	Q	130,000				

Table S1. Model architecture details and checkpoint selections. Each row corresponds to a distinct configuration of a pretrained model, varying by model size (in millions of parameters), training data size (Full, Half, or Quarter), and chosen checkpoint (in training steps).

bioRxiv preprint doi: https://doi.org/10.1101/2025.09.02.673765; this version posted November 5, 2025. The copyright holder for this preprint (which was not certified by peer review) is the author/funder, who has granted bioRxiv a license to display the preprint in perpetuity. It is made available under aCC-BY 4.0 International license.

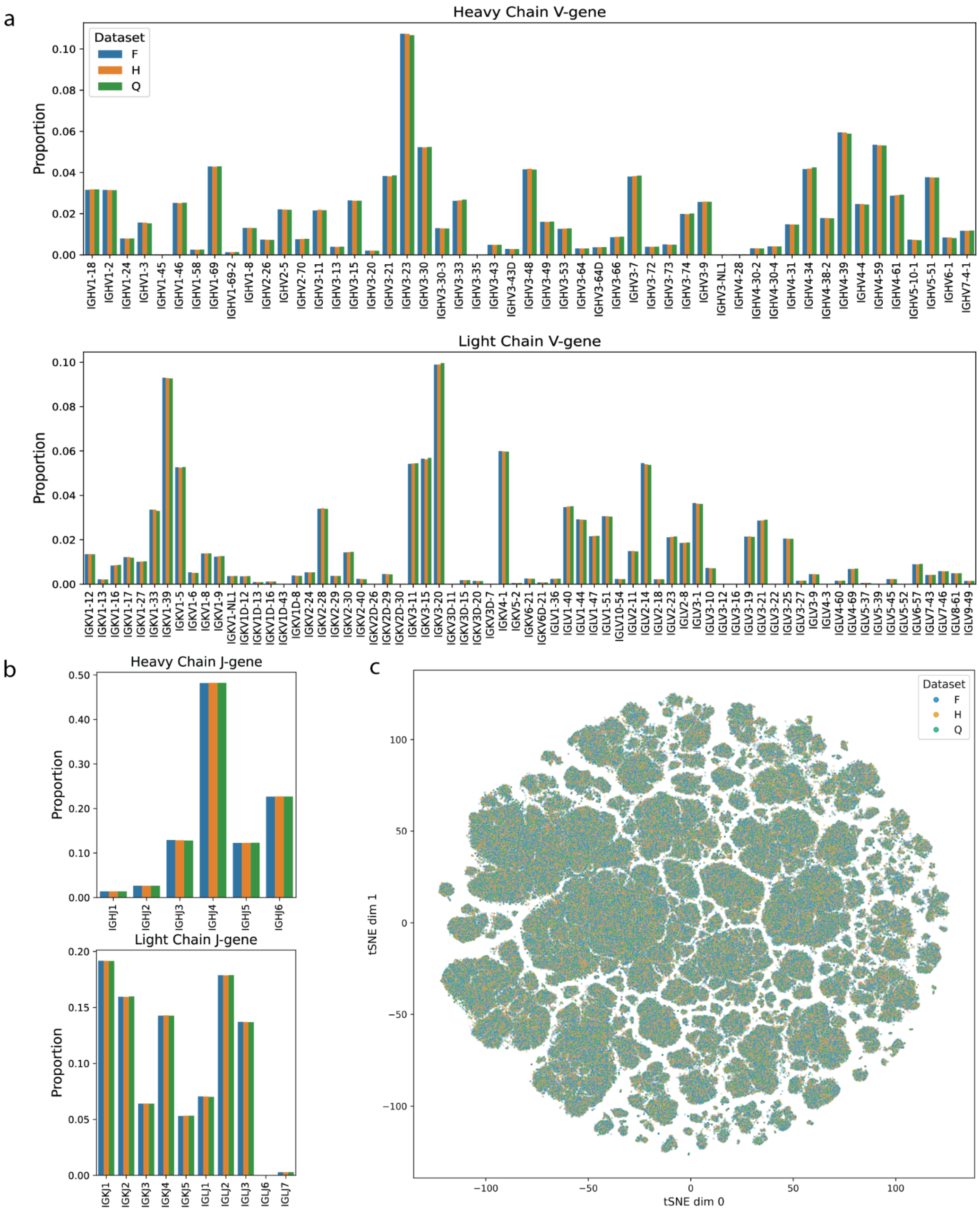

Figure S1. Distribution of V- and J-gene usage and sequence diversity across datasets. (a) V-gene usage across training-set splits. Grouped bar plots show the proportion of sequences in each dataset with a particular heavy-chain V (top) or light-chain V (bottom) gene. (b) J-gene usage across training-set splits. Grouped bar plots show the proportion of sequences in each dataset with a particular heavy-chain J (top) or light-chain J (bottom) gene. (c) Sequence diversity visualized using a t-SNE projection of ESM-2-650M embeddings, with each point representing a single paired sequence. All training sets exhibit comparable diversity.

bioRxiv preprint doi: https://doi.org/10.1101/2025.09.02.673765; this version posted November 5, 2025. The copyright holder for this preprint (which was not certified by peer review) is the author/funder, who has granted bioRxiv a license to display the preprint in perpetuity. It is made available under aCC-BY 4.0 International license.

Feature	Training Splits	# of Categories	χ^2 Statistic	χ^2 p-value
Heavy Chain V-gene	Quarter vs Half	51	20.079451	0.999950
	Half vs Full	51	17.889161	0.999992
	Quarter vs Full	51	37.792155	0.897789
Heavy Chain J-gene	Quarter vs Half	6	1.066540	0.957022
	Half vs Full	6	2.090143	0.836537
	Quarter vs Full	6	3.341937	0.647428
Heavy Chain VJ-gene usage	Quarter vs Half	299	107.238132	1.000000
	Half vs Full	301	101.794289	1.000000
	Quarter vs Full	301	198.955262	0.999999
Light Chain V-gene	Quarter vs Half	69	22.444984	1.000000
	Half vs Full	69	25.491875	0.999999
	Quarter vs Full	69	44.962355	0.986015
Light Chain J-gene	Quarter vs Half	10	2.113995	0.989534
	Half vs Full	10	1.188426	0.998866
	Quarter vs Full	10	4.035732	0.909045
Light Chain VJ-gene usage	Quarter vs Half	302	94.901586	1.000000
	Half vs Full	304	100.277863	1.000000
	Quarter vs Full	304	179.888776	1.000000

Table S2. χ^2 tests of gene-usage distributions across training-set splits. Pairwise comparisons between datasets (Q vs H, H vs F, Q vs F) of heavy and light chain V, J, and V/J gene-usage distribution. Comparisons are reported with the number of categories tested, the χ^2 statistic, and the corresponding p-value.

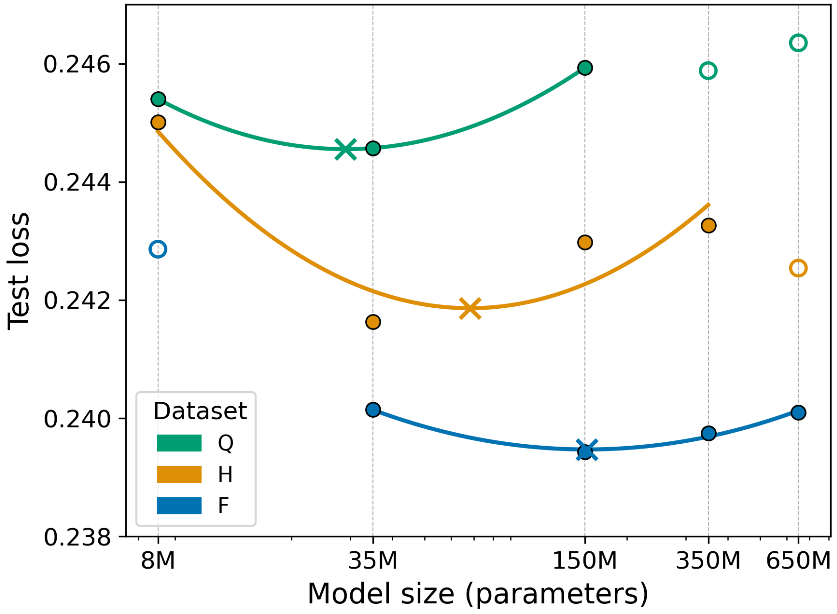

bioRxiv preprint doi: https://doi.org/10.1101/2025.09.02.673765; this version posted November 5, 2025. The copyright holder for this preprint (which was not certified by peer review) is the author/funder, who has granted bioRxiv a license to display the preprint in perpetuity. It is made available under aCC-BY 4.0 International license.

Figure S2. Cross-entropy loss curves with all evaluated points for each dataset size. Model sizes were log-transformed and evaluated to generate FixedData profiles. Circles (O) represent the mean loss averaged over 10 donor datasets for each model–data size combination, while crosses (X) mark the fitted curve minimum corresponding to the lowest loss. In this figure, all evaluated mean cross entropy losses are displayed, with outlier points shown as unfilled circles.

Dataset	Model size (M)	Average loss
**Q**	8	0.2454
	35	0.2445
	150	0.2459
	350	0.2458
	650	0.2463
**H**	8	0.2450
	35	0.2416
	150	0.2429
	350	0.2432
	650	0.2425
**F**	8	0.2428
	35	0.2401
	150	0.2394
	350	0.2397
	650	0.2401

Table S3. Average cross-entropy loss during evaluating models across different scales that were evaluated using a masked language modeling (MLM) objective on data from 10 distinct donors.

Region	Condition	Model size (M)	Loss	Perplexity
**CDRH3**	**Mutated**	8	1.2026	3.4237
		35	1.1513	3.2293
		150	1.0970	3.0614
		350	1.1009	3.0585
		650	1.1502	3.2378
	**Unmutated**	8	0.2709	1.3149
		35	0.2797	1.3152
		150	0.2694	1.3136
		350	0.2643	1.3092
		650	0.2754	1.3239

Table S4. CDRH3 prediction metrics for all full data models. Median cross entropy loss and median perplexity of models trained on the full dataset for per-residue prediction in the CDRH3. Metrics are shown across varying model sizes (in millions of parameters) and grouped by mutated and germline (unmutated) sequence categories.

bioRxiv preprint doi: https://doi.org/10.1101/2025.09.02.673765; this version posted November 5, 2025. The copyright holder for this preprint (which was not certified by peer review) is the author/funder, who has granted bioRxiv a license to display the preprint in perpetuity. It is made available under aCC-BY 4.0 International license.

Model 1	Model 2	Pairs	t-test	P-value	Sig
8	35	240829	3.961	0.001	*
8	150	240829	2.917	0.017	*
8	350	240829	2.793	0.017	*
8	650	240829	1.468	0.237	
35	150	240829	-1.012	0.346	
35	350	240829	-1.132	0.322	
35	650	240829	-2.517	0.030	*
150	350	240829	-0.121	0.904	
150	650	240829	-1.483	0.237	
350	650	240829	-1.360	0.248	

Table S5. Paired t-test across model sizes for D-segment prediction on the CDRH3 region. Each comparison reports the t-statistic, Benjamini–Hochberg corrected p-value, and significance level for paired evaluations across models. Asterisks indicate statistically significant differences between models.

Classification	Dataset	Model size (M)	Accuracy	AUC	AUPR	MCC	F1 Score
CoV vs. Healthy Donors	Quarter	8	0.6740 (± 0.0034)	0.7362 (± 0.0025)	0.7253 (± 0.0024)	0.3504 (± 0.0067)	0.6923 (± 0.0028)
		35	0.6820 (± 0.0023)	0.7445 (± 0.0024)	0.7354 (± 0.0018)	0.3643 (± 0.0045)	0.6866 (± 0.0026)
		150	0.6884 (± 0.0042)	0.7541 (± 0.0037)	0.7520 (± 0.0037)	0.3773 (± 0.0083)	0.6954 (± 0.0046)
		350	**0.6944** (± 0.0021)	**0.7673** (± 0.0015)	**0.7699** (± 0.0016)	**0.3900** (± 0.0041)	**0.7048** (± 0.0025)
		650	0.6909 (± 0.0029)	0.7608 (± 0.0020)	0.7656 (± 0.0016)	0.3827 (± 0.0059)	0.6981 (± 0.0053)
	Half	8	0.6732 (± 0.0035)	0.7411 (± 0.0031)	0.7350 (± 0.0033)	0.3520 (± 0.0071)	0.6998 (± 0.0032)
		35	0.6869 (± 0.0028)	0.7544 (± 0.0025)	0.7487 (± 0.0038)	0.3756 (± 0.0057)	0.7014 (± 0.0033)
		150	0.6974 (± 0.0036)	0.7667 (± 0.0023)	0.7667 (± 0.0018)	0.3955 (± 0.0072)	0.7057 (± 0.0043)
		350	**0.7042** (± 0.0023)	**0.7765** (± 0.0017)	**0.7818** (± 0.0027)	**0.4088** (± 0.0045)	**0.7093** (± 0.0033)
		650	0.6990 (± 0.0034)	0.7697 (± 0.0023)	0.7716 (± 0.0031)	0.3988 (± 0.0068)	0.7067 (± 0.0043)
Influenza vs. CoV vs. Healthy Donor	Quarter	8	0.6062 (± 0.0051)	-	-	0.4169 (± 0.0067)	0.6058 (± 0.0049)
		35	0.5769 (± 0.0109)	-	-	0.3699 (± 0.0159)	0.5751 (± 0.0119)
		150	0.6444 (± 0.0078)	-	-	0.4694 (± 0.0118)	0.6448 (± 0.0074)
		350	**0.6467** (± 0.0057)	-	-	**0.4731** (± 0.0076)	**0.6462** (± 0.0059)
		650	0.6353 (± 0.0041)	-	-	0.4558 (± 0.0062)	0.6364 (± 0.0036)
	Half	8	0.5928 (± 0.0055)	-	-	0.3986 (± 0.0056)	0.5894 (± 0.0060)
		35	0.6253 (± 0.0083)	-	-	0.4458 (± 0.0116)	0.6230 (± 0.0093)
		150	0.6476 (± 0.0084)	-	-	0.4756 (± 0.0117)	0.6481 (± 0.0090)
		350	**0.6610** (± 0.0043)	-	-	**0.4949** (± 0.0067)	**0.6619** (± 0.0038)
		650	0.6576 (± 0.0026)	-	-	0.4888 (± 0.0036)	0.6597 (± 0.0026)

Table S6: Performance of antibody specificity classification models across AbLMs trained on Dataset-H and Dataset-Q. Binary classification results for distinguishing CoV-specific antibodies from healthy donor sequences. Three-way classification results differentiating Influenza-specific, CoV-specific, and healthy donor antibodies. For each dataset size, the best model is indicated in **bold** per metric.

bioRxiv preprint doi: https://doi.org/10.1101/2025.09.02.673765; this version posted November 5, 2025. The copyright holder for this preprint (which was not certified by peer review) is the author/funder, who has granted bioRxiv a license to display the preprint in perpetuity. It is made available under aCC-BY 4.0 International license.

Classification	Dataset	Model Size (M)	Accuracy	AUC	AUPR	MCC	F1 Score
Native vs. Shuffled Pairing	Quarter	8	0.6540 (± 0.0024)	0.6944 (± 0.0029)	0.7187 (± 0.0028)	0.3180 (± 0.0051)	0.6050 (± 0.0025)
		35	0.6663 (± 0.0022)	0.7063 (± 0.0031)	0.7432 (± 0.0032)	0.3485 (± 0.0048)	0.6081 (± 0.0021)
		150	0.6664 (± 0.0010)	0.7065 (± 0.0017)	0.7367 (± 0.0019)	0.3479 (± 0.0021)	0.6096 (± 0.0015)
		350	0.6534 (± 0.0017)	0.7020 (± 0.0023)	0.7217 (± 0.0022)	0.3200 (± 0.0036)	0.5961 (± 0.0020)
		650	0.6174 (± 0.0012)	0.6644 (± 0.0020)	0.6726 (± 0.0015)	0.2424 (± 0.0026)	0.5628 (± (0.0012)
	Half	8	0.6480 (± 0.0021	0.6857 (± 0.0028)	0.6986 (± 0.0028)	0.2999 (± 0.0043)	0.6169 (± 0.0023)
		35	0.6650 (±0.0023	0.7086 (± 0.0023)	0.7488 (± 0.0024)	0.3519 (± 0.0047)	0.5947 (± 0.0031)
		150	0.6701 (± 0.0016)	0.7167 (± 0.0016)	0.7539 (± 0.0014)	0.3568 (± 0.0035)	0.6116 (± 0.0017)
		350	0.6773 (± 0.0021)	0.7316 (± 0.0017)	0.7653 (± 0.0018)	0.3762 (± 0.0044)	0.6126 (± 0.0028)
		650	0.6429 (± 0.0021)	0.6919 (± 0.0020)	0.7164 (± 0.0017)	0.2920 (± 0.0046)	0.6022 (± 0.0016)
	Full	8	0.6573 (± 0.0007	0.6994 (± 0.0024)	0.7414 (± 0.0021)	0.3323 (± 0.0019)	0.5914 (± 0.0012)
		35	0.6832 (± 0.0009)	0.7239 (± 0.0008)	0.7695 (± 0.0010)	0.3998 (± 0.0021)	0.6041 (± 0.0017)
		150	0.6696 (± 0.0023)	0.7200 (± 0.0027)	0.7616 (± 0.0021)	0.3519 (± 0.0052)	0.6187 (± 0.0018)
		350	**0.7046** (± 0.0017)	**0.7618** (± 0.0018)	**0.8056** (± 0.0014)	**0.4399** (± 0.0037)	**0.6381** (± 0.0021)
		650	0.6917 (± 0.0020)	0.7438 (± 0.0020)	0.7868 (± 0.0017)	0.4107 (± 0.0038)	0.6242 (± 0.0030)

Table S7. Detailed classification results for pair classification. Performance metrics are presented for models ranging from 8M to 650M parameters, evaluated across Dataset-F, Dataset-H, and Dataset-Q. The best-performing model for each metric across all datasets is shown in **bold**, while the top-performing model for each metric within individual datasets is underlined.

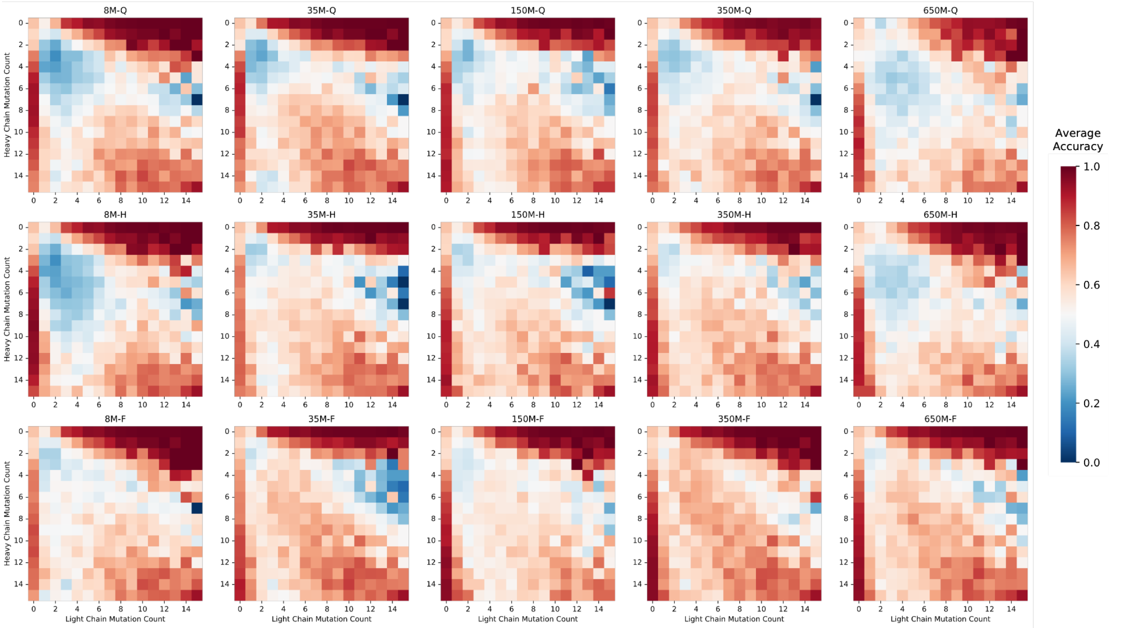

Figure S3. Classification accuracy across all model sizes and data scales for pair classification. Heatmaps show average classification accuracy for all models for different combinations of chain-specific mutation counts. Darker red values indicate higher accuracy, while lighter blue values indicate lower accuracy.
