# Supplementary material for: Data-optimal scaling of paired antibody language models
Source: bioRxiv. 2025 Sep 6:2025.09.02.673765. Preprint. [Version 1] doi: 10.1101/2025.09.02.673765 (PMC12424686; doi:10.1101/2025.09.02.673765)
Supplement: Supplementary file 1 [file NIHPP2025.09.02.673765v1-supplement-1.pdf]

bioRxiv preprint doi: https://doi.org/10.1101/2025.09.02.673765; this version posted September 6, 2025. The copyright holder for this preprint (which was not certified by peer review) is the author/funder, who has granted bioRxiv a license to display the preprint in perpetuity. It is made available under aCC-BY 4.0 International license.

SUPPLEMENTARY INFORMATION

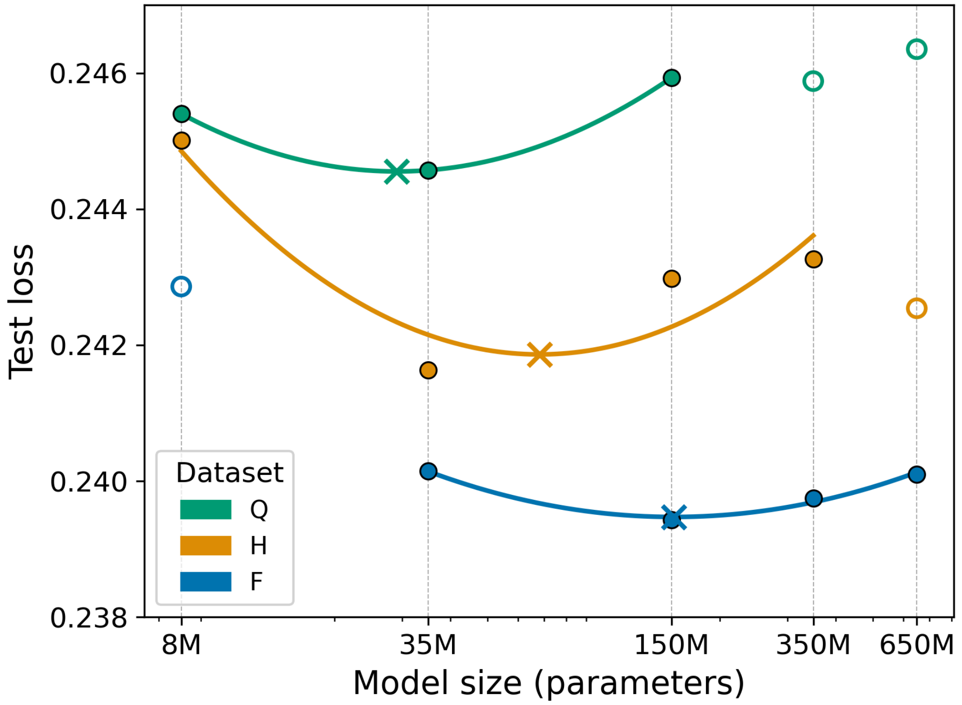

Figure S1. Cross-entropy loss curves with all evaluated points for each dataset size. Model sizes were log-transformed and evaluated to generate FixedData profiles. Circles (O) represent the mean loss averaged over 10 donor datasets for each model–data size combination, while crosses (X) mark the fitted curve minimum corresponding to the lowest loss. In this figure, all evaluated mean cross entropy losses are displayed, with outlier points shown as unfilled circles.

bioRxiv preprint doi: https://doi.org/10.1101/2025.09.02.673765; this version posted September 6, 2025. The copyright holder for this preprint (which was not certified by peer review) is the author/funder, who has granted bioRxiv a license to display the preprint in perpetuity. It is made available under aCC-BY 4.0 International license.

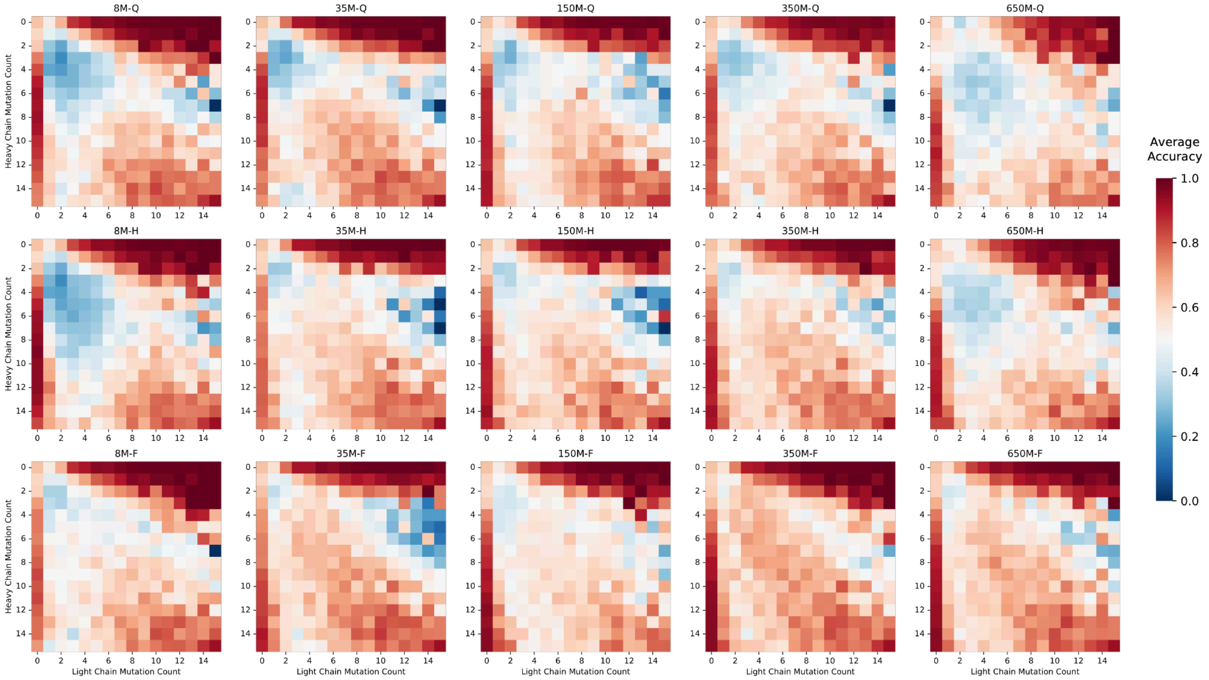

Figure S2. Classification accuracy across all model sizes and data scales for pair classification. Heatmaps show average classification accuracy for all models for different combinations of chain-specific mutation counts. Darker red values indicate higher accuracy, while lighter blue values indicate lower accuracy.

bioRxiv preprint doi: https://doi.org/10.1101/2025.09.02.673765; this version posted September 6, 2025. The copyright holder for this preprint (which was not certified by peer review) is the author/funder, who has granted bioRxiv a license to display the preprint in perpetuity. It is made available under aCC-BY 4.0 International license.

Model size (M)	Dataset	Checkpoint step	Transformer layers	Attention heads	Hidden size	Intermediate size
8	F	500,000	6	20	320	1280
	H	435,000				
	Q	425,000				
35	F	500,000	12	20	480	1920
	H	430,000				
	Q	240,000				
150	F	500,000	30	20	640	2560
	H	330,000				
	Q	165,000				
350	F	500,000	32	20	960	3840
	H	300,000				
	Q	155,000				
650	F	395,000	33	20	1280	5120
	H	330,000				
	Q	130,000				

Table S1. Model architecture details and checkpoint selections. Each row corresponds to a distinct configuration of a pretrained model, varying by model size (in millions of parameters), training data size (Full, Half, or Quarter), and chosen checkpoint (in training steps).

bioRxiv preprint doi: https://doi.org/10.1101/2025.09.02.673765; this version posted September 6, 2025. The copyright holder for this preprint (which was not certified by peer review) is the author/funder, who has granted bioRxiv a license to display the preprint in perpetuity. It is made available under aCC-BY 4.0 International license.

Dataset	Model size (M)	Average loss
Q	8	0.2454
	35	0.2445
	150	0.2459
	350	0.2458
	650	0.2463
H	8	0.2450
	35	0.2416
	150	0.2429
	350	0.2432
	650	0.2425
F	8	0.2428
	35	0.2401
	150	0.2394
	350	0.2397
	650	0.2401

Table S2. Average cross-entropy loss during evaluating models across different scales that were evaluated using a masked language modeling (MLM) objective on data from 10 distinct donors.

bioRxiv preprint doi: https://doi.org/10.1101/2025.09.02.673765; this version posted September 6, 2025. The copyright holder for this preprint (which was not certified by peer review) is the author/funder, who has granted bioRxiv a license to display the preprint in perpetuity. It is made available under aCC-BY 4.0 International license.

Region	Condition	Model size (M)	Loss	Perplexity
CDRH3	Mutated	8	1.2026	3.4237
		35	1.1513	3.2293
		150	1.0970	3.0614
		350	1.1009	3.0585
		650	1.1502	3.2378
	Unmutated	8	0.2709	1.3149
		35	0.2797	1.3152
		150	0.2694	1.3136
		350	0.2643	1.3092
		650	0.2754	1.3239

Table S3. CDRH3 prediction metrics for all full data models. Median cross entropy loss and median perplexity of models trained on the full dataset for per-residue prediction in the CDRH3. Metrics are shown across varying model sizes (in millions of parameters) and grouped by mutated and germline (unmutated) sequence categories.

bioRxiv preprint doi: https://doi.org/10.1101/2025.09.02.673765; this version posted September 6, 2025. The copyright holder for this preprint (which was not certified by peer review) is the author/funder, who has granted bioRxiv a license to display the preprint in perpetuity. It is made available under aCC-BY 4.0 International license.

Classification	Dataset	Model size (M)	Accuracy	AUC	AUPR	MCC	F1 Score
CoV vs. Healthy Donors	Quarter	8	0.6740 (± 0.0034)	0.7362 (± 0.0025)	0.7253 (± 0.0024)	0.3504 (± 0.0067)	0.6923 (± 0.0028)
		35	0.6820 (± 0.0023)	0.7445 (± 0.0024)	0.7354 (± 0.0018)	0.3643 (± 0.0045)	0.6866 (± 0.0026)
		150	0.6884 (± 0.0042)	0.7541 (± 0.0037)	0.7520 (± 0.0037)	0.3773 (± 0.0083)	0.6954 (± 0.0046)
		350	**0.6944** (± 0.0021)	**0.7673** (± 0.0015)	**0.7699** (± 0.0016)	**0.3900** (± 0.0041)	**0.7048** (± 0.0025)
		650	0.6909 (± 0.0029)	0.7608 (± 0.0020)	0.7656 (± 0.0016)	0.3827 (± 0.0059)	0.6981 (± 0.0053)
	Half	8	0.6732 (± 0.0035)	0.7411 (± 0.0031)	0.7350 (± 0.0033)	0.3520 (± 0.0071)	0.6998 (± 0.0032)
		35	0.6869 (± 0.0028)	0.7544 (± 0.0025)	0.7487 (± 0.0038)	0.3756 (± 0.0057)	0.7014 (± 0.0033)
		150	0.6974 (± 0.0036)	0.7667 (± 0.0023)	0.7667 (± 0.0018)	0.3955 (± 0.0072)	0.7057 (± 0.0043)
		350	**0.7042** (± 0.0023)	**0.7765** (± 0.0017)	**0.7818** (± 0.0027)	**0.4088** (± 0.0045)	**0.7093** (± 0.0033)
		650	0.6990 (± 0.0034)	0.7697 (± 0.0023)	0.7716 (± 0.0031)	0.3988 (± 0.0068)	0.7067 (± 0.0043)
Influenza vs. CoV vs. Healthy Donor	Quarter	8	0.6062 (± 0.0051)	-	-	0.4169 (± 0.0067)	0.6058 (± 0.0049)
		35	0.5769 (± 0.0109)	-	-	0.3699 (± 0.0159)	0.5751 (± 0.0119)
		150	0.6444 (± 0.0078)	-	-	0.4694 (± 0.0118)	0.6448 (± 0.0074)
		350	**0.6467** (± 0.0057)	-	-	**0.4731** (± 0.0076)	**0.6462** (± 0.0059)
		650	0.6353 (± 0.0041)	-	-	0.4558 (± 0.0062)	0.6364 (± 0.0036)
	Half	8	0.5928 (± 0.0055)	-	-	0.3986 (± 0.0056)	0.5894 (± 0.0060)
		35	0.6253 (± 0.0083)	-	-	0.4458 (± 0.0116)	0.6230 (± 0.0093)
		150	0.6476 (± 0.0084)	-	-	0.4756 (± 0.0117)	0.6481 (± 0.0090)
		350	**0.6610** (± 0.0043)	-	-	**0.4949** (± 0.0067)	**0.6619** (± 0.0038)
		650	0.6576 (± 0.0026)	-	-	0.4888 (± 0.0036)	0.6597 (± 0.0026)

Table S4: Performance of antibody specificity classification models across AbLMs trained on Dataset-H and Dataset-Q. Binary classification results for distinguishing CoV-specific antibodies from healthy donor sequences. Three-way classification results differentiating Influenza-specific, CoV-specific, and healthy donor antibodies. For each dataset size, the best model is indicated in **bold** per metric.

bioRxiv preprint doi: https://doi.org/10.1101/2025.09.02.673765; this version posted September 6, 2025. The copyright holder for this preprint (which was not certified by peer review) is the author/funder, who has granted bioRxiv a license to display the preprint in perpetuity. It is made available under aCC-BY 4.0 International license.

Classification	Dataset	Model Size (M)	Accuracy	AUC	AUPR	MCC	F1 Score
Native vs. Shuffled Pairing	Quarter	8	0.6540 (± 0.0024)	0.6944 (± 0.0029)	0.7187 (± 0.0028)	0.3180 (± 0.0051)	0.6050 (± 0.0025)
		35	0.6663 (± 0.0022)	0.7063 (± 0.0031)	0.7432 (± 0.0032)	0.3485 (± 0.0048)	0.6081 (± 0.0021)
		150	0.6664 (± 0.0010)	0.7065 (± 0.0017)	0.7367 (± 0.0019)	0.3479 (± 0.0021)	0.6096 (± 0.0015)
		350	0.6534 (± 0.0017)	0.7020 (± 0.0023)	0.7217 (± 0.0022)	0.3200 (± 0.0036)	0.5961 (± 0.0020)
		650	0.6174 (± 0.0012)	0.6644 (± 0.0020)	0.6726 (± 0.0015)	0.2424 (± 0.0026)	0.5628 (± (0.0012)
	Half	8	0.6480 (± 0.0021	0.6857 (± 0.0028)	0.6986 (± 0.0028)	0.2999 (± 0.0043)	0.6169 (± 0.0023)
		35	0.6650 (±0.0023	0.7086 (± 0.0023)	0.7488 (± 0.0024)	0.3519 (± 0.0047)	0.5947 (± 0.0031)
		150	0.6701 (± 0.0016)	0.7167 (± 0.0016)	0.7539 (± 0.0014)	0.3568 (± 0.0035)	0.6116 (± 0.0017)
		350	0.6773 (± 0.0021)	0.7316 (± 0.0017)	0.7653 (± 0.0018)	0.3762 (± 0.0044)	0.6126 (± 0.0028)
		650	0.6429 (± 0.0021)	0.6919 (± 0.0020)	0.7164 (± 0.0017)	0.2920 (± 0.0046)	0.6022 (± 0.0016)
	Full	8	0.6573 (± 0.0007	0.6994 (± 0.0024)	0.7414 (± 0.0021)	0.3323 (± 0.0019)	0.5914 (± 0.0012)
		35	0.6832 (± 0.0009)	0.7239 (± 0.0008)	0.7695 (± 0.0010)	0.3998 (± 0.0021)	0.6041 (± 0.0017)
		150	0.6696 (± 0.0023)	0.7200 (± 0.0027)	0.7616 (± 0.0021)	0.3519 (± 0.0052)	0.6187 (± 0.0018)
		350	**0.7046** (± 0.0017)	**0.7618** (± 0.0018)	**0.8056** (± 0.0014)	**0.4399** (± 0.0037)	**0.6381** (± 0.0021)
		650	0.6917 (± 0.0020)	0.7438 (± 0.0020)	0.7868 (± 0.0017)	0.4107 (± 0.0038)	0.6242 (± 0.0030)

Table S5. Detailed classification results for pair classification. Performance metrics are presented for models ranging from 8M to 650M parameters, evaluated across Dataset-F, Dataset-H, and Dataset-Q. The best-performing model for each metric across all datasets is shown in **bold**, while the top-performing model for each metric within individual datasets is underlined.
